# Torticollis

**Published:** 1889-08

**Authors:** B. Simmons

**Affiliations:** Sydney, N. S. W.


### TORTICOLLIS.

On referring to Dr. Jefferson Guernsey's most valuable card on *Diphtheria*, I see that Lachnanthes is the only remedy named for the symptom "neck drawn to one side," and as we have not seen many verifications where this symptom has been present, it occurred to me that a description of one or two cases that have been under my medical care might be useful to record.

CASE I.—E. S., aged seven years, pale face, blue eyes, light hair, was attacked with feverishness, restlessness, *neck spasmodically drawn to the right side*, flushed face, starting when asleep. Bell.$^{200}$ every four hours was followed by improvement of the fever-flushed face and starting, but the distortion of the neck remained the same. I now learned for the first time that *during sleep* the child frequently uttered *sharp, piercing screams.* Apis mell.$^{200}$ every four hours was administered. A rapid improvement now commenced. In twenty-four hours I could observe that the *morale* of the child was better, the neck less crooked; better sleep, with fewer screams. I continued the same remedy at longer intervals for several days, during which time the child greatly improved in health. Within forty-eight hours of giving Apis the head was perfectly straight. I learned from the relatives of the child that for several months after this attack the little patient had never been observed in such excellent health. The promptitude with which this medicine removed the torticollis satisfied me that in cases of wry neck it is a medicine that should be thought of when brain symptoms are present. I may add that the distortion of the neck had existed a week previous to my seeing it, the little fellow being treated during that time for rheumatism by embrocations of various kinds, all of which had no effect.

CASE II.—E. F., aged six years, extremely delicate from birth, was attacked with diphtheritic sore throat, *worse on the right side*, neck swollen, and *spasmodically drawn to the right side, intolerance of light*, intense fever, rapid pulse and prostration, aversion to all kinds of food except oranges, the juice of which was taken freely. Lyc.$^{200}$ every two hours was prescribed; improvement was observable in twenty-four hours. The child made a good recovery under this medicine alone. I may here remark that in the throat cases that require Lyc., *intolerance of light* is often a marked symptom—that is, so far as my experience goes. Orange juice as a nutriment is, I believe, most valuable, especially when the patient craves it, though it is well known that in some cases of croup it is injurious. Of its sustaining qualities I witnessed a remarkable instance in the early years of my practice. A delicate young girl, aged thirteen, took putrid scarlet fever; she was extremely ill and *bled from every orifice of the body.* For ten days she existed on orange juice alone, all other nourishment being obstinately refused. She made a slow but perfect recovery. Crotalus was no doubt the remedy indicated, but at that time I was unacquainted with its virtues.

CASE III.—A. C., aged six, was brought to me with his head spasmodically drawn to the right side. The distortion had existed about a week and commenced when on shipboard. His father informed me that he had always been a delicate child, but I was unable to obtain any characteristic symptoms to guide me in selecting a remedy. I accordingly gave him Lachnan.$^{30}$ every four hours. I saw him again in two days, but no improvement had taken place. On examining the right side of the neck I found the cervical glands much enlarged and extremely tender to the touch, and as he was a nervous, excitable boy I gave Bell.$^{200}$ but without effect. Lyc.$^{200}$ every four hours proved to be the curative remedy.

Many years ago, at the request of my friend Dr. Drysdale, of Liverpool, I translated from a French journal the particulars of a most interesting case of torticollis which had existed for a long time in the person of a Roman Catholic priest. All attempts to relieve the spasm were fruitless, until Lyc. was administered, when a cure resulted. The case was published in the *British Journal of Homœopathy*, but I regret that I cannot remember either the potency administered or the date of the journal. Remembering this cure encouraged me in selecting Lyc. in the diphtheritic case before given, and also in giving the same medicine when Bell. and Lachnan. had failed. In the three instances that occurred in my own practice the patients were boys, delicate, light-complexioned, fair hair and of nearly the same age, the neck also being drawn to the *right side.*

SYDNEY, N. S. W. B. SIMMONS, M. D.

